# Invest more and die faster: The life history of a parasite on intensive farms

**DOI:** 10.1111/eva.12488

**Published:** 2017-06-21

**Authors:** Adèle Mennerat, Mathias Stølen Ugelvik, Camilla Håkonsrud Jensen, Arne Skorping

**Affiliations:** ^1^ Department of Biology University of Bergen Bergen Norway; ^2^ Ecologie et Dynamique des Systèmes Anthropisés (FRE 3498) CNRS/Université de Picardie Jules Verne Amiens France

**Keywords:** human‐induced evolution, intensive aquaculture, *Lepeophtheirus salmonis*, life history trade‐offs, *Salmo salar*

## Abstract

Organisms are expected to respond to alterations in their survival by evolutionary changes in their life history traits. As agriculture and aquaculture have become increasingly intensive in the past decades, there has been growing interest in their evolutionary effects on the life histories of agri‐ and aquacultural pests, parasites, and pathogens. In this study, we used salmon lice (*Lepeophtheirus salmonis*) to explore how modern farming might have affected life history evolution in parasites. We infected salmon hosts with lice from either farmed or unfarmed locations, and monitored life history traits of those parasites in laboratory conditions. Our results show that compared to salmon lice from areas unaffected by salmon farming, those from farmed areas produced more eggs in their first clutch, and less eggs later on; they achieved higher infestation intensities in early adulthood, but suffered higher adult mortality. These results suggest that salmon lice on farms may have been selected for increased investment in early reproduction, at the expense of later fecundity and survival. This call for further empirical studies of the extent to which farming practices may alter the virulence of agricultural parasites.

## 1 INTRODUCTION

Agri‐ and aquacultural practices have become increasingly intensive in the past decades, which represents a change in the ecology of domesticated species (Turcotte, Araki, Karp, Poveda, & Whitehead, 2017). In parallel, there has been growing concern that this may also cause evolutionary changes in the life histories of agricultural pests and diseases (Kennedy et al., 2016; Lebarbenchon, Brown, Poulin, Gauthier‐Clerc, & Thomas, 2008; Mennerat, Nilsen, Ebert, & Skorping, 2010; Rogalski, Gowler, Shaw, Hufbauer, & Duffy, 2016; Rozins & Day, 2016). High local densities of animals or plants, reduced genetic variation, and breeding of stocks for a high output usually characterize intensive agricultural systems. While farming‐induced evolution of parasite virulence is being explored in a number of reviews and theoretical studies (e.g. Kennedy et al., 2016; Mennerat et al., 2010; Rozins & Day, 2016), empirical studies so far have mainly focused on short‐term issues like drug resistance, producing new treatments, and reducing the economic losses caused by parasites (but see Leignel & Cabaret, 2001; Pulkkinen et al., 2010; Sundberg et al., 2016).

For iteroparous organisms, a well‐established cost of current reproduction is a decrease in future (or residual) reproductive value, which is a combination of future fecundity and survival (Minchella & Loverde, 1981; Roff, 2002; Stearns, 1992). Therefore, an individual that restrains its current reproduction will have more resources available to invest in growth and survival, and thereby benefit from higher future fecundity. A second trade‐off is that between the number of offspring and the amount of resources invested into each of them (e.g., Smith & Fretwell, 1974). As the amount of resources available for reproduction is limited, organisms have the option of either making few, high quality offspring or many offspring of a lower quality. How these trade‐offs are resolved depends on the shape of the survival curve (Stearns, 1992); both free‐living and parasitic species are, therefore, expected to respond to alterations in their survival (including human‐induced) by evolutionary changes in their life history traits (Skorping, Jensen, Mennerat, & Högstedt, 2016).

One of the most rapidly growing forms of food production in recent years is intensive aquaculture. The Norwegian Atlantic salmon (*Salmo salar*) mariculture stocks alone have increased from about 160 thousand to more than 720 thousand metric tonnes from 1994 to 2015, while the number of licensed farms has only increased from 811 to 974, which means that both density and turnover rate of fish on each farm have increased dramatically (Norwegian Directorate of Fisheries, 2015). As with other forms of intensive food production, fish in intensive aquaculture facilities are usually densely stocked and kept in monocultures. These unnaturally high population sizes do not only translate into more potential hosts for the parasites but can also be viewed as a highly predictable year‐round resource as fish with a migratory behavior are kept in cages (Kennedy et al., 2016; Mennerat et al., 2010). In addition, compared with other farming practices (e.g., swine or chicken domestication) salmon mariculture is relatively recent, and there still exist areas both untouched by these food production practices and relatively isolated from farmed areas. This makes salmon farming an especially good system for studying parasite evolutionary responses to human‐induced changes in host ecology.

The ectoparasitic sea lice (Caligidae) that feed on the skin, mucus, and blood of salmonid fish are among the most widespread marine parasites in salmon aquaculture (Costello, 2006). Within this family, it is the salmon louse (*Lepeophtheirus salmonis*) that has received most attention because of the problems it causes for both the industry and wild salmonid populations. Its life cycle consists of eight developmental stages (Hamre et al., 2013), and calculations indicate that about seven generations can be produced in 1 year (Whelan, 2010). The salmon louse has an iteroparous life cycle, and up to 11 successive pairs of egg strings (i.e.*,* clutches) have been reported (Heuch, Nordhagen, & Schram, 2000; Mennerat et al., 2012).

Before mariculture started and in areas still unaffected by aquaculture, the salmon louse was depending on migrating salmon that sporadically came into the fjords and on their offspring swimming out, as well as on the resident sea trout populations in coastal areas. The salmon louse's iteroparous life cyle can therefore be viewed as a bet‐hedging strategy to an unpredictable host resource (e.g., Beaumont, Gallie, Kost, Ferguson, & Rainey, 2009). Salmon lice epidemics started to be reported soon after cage culture began, in the 1960's in Norway, the 1970's in Scotland, and in the 1980's in North America, and were attributed to the significantly increased host resource in the sea (Mennerat et al., 2010; Pike & Wadsworth, 2000). In addition to a high host density “farmed” lice are also exposed to frequent host culling and chemical treatments (Denholm et al., 2002), both of which lead to shorter life expectancies of adults on the host (Mennerat et al., 2010).

The differences between the environments of “farmed” and “wild” salmon lice may select for different strategies: Infective stages of “farmed” lice have a high probability of finding a host, but due to treatments and culling of hosts the prospect of a long adult life once infection has been achieved is rather poor. For “wild” lice chances of infecting a host are much lower, but life expectancy after infection may be better. This shift in selection from wild to farmed host populations may have favored increased investment in current reproduction (Mennerat et al., 2010), which can be achieved by increasing early fecundity and/or by producing eggs of a better quality.

In this study, we focus on the double hypothesis that (i) “farmed” lice have been selected to achieve higher reproductive output soon after maturity, and invest more than “wild” lice in offspring quality, and (ii) such a shift toward increased current reproduction comes at the cost of decreased fitness later in life. In salmon lice, the quality of offspring (i.e.*,* larvae) is expressed in their ability to find a suitable host, infect it, and develop on it until adulthood. We investigated these predictions by comparing infection success, fecundity, and adult survival of salmon lice sampled from relatively isolated areas where there has never been any salmon farming, to lice coming from areas where salmon have been intensively farmed for several decades.

## 2 MATERIALS AND METHODS

### 2.1 *Lepeophtheirus salmonis* sample

**Figure 1:**
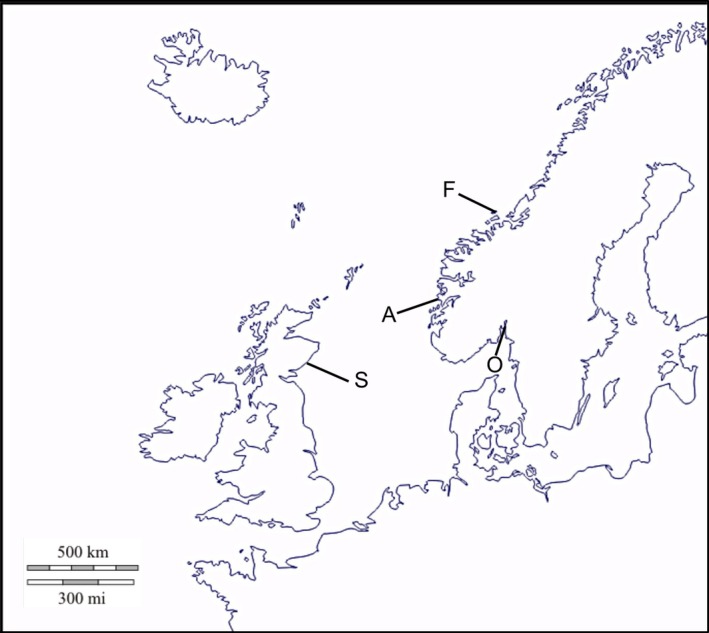
Map of Northern Europe showing the sampling locations of the four study groups of salmon lice. A: Austevoll (farmed); F: Frøya (farmed); O: Oslofjord (unfarmed); S: Scotland (unfarmed)

In this study, we monitored the life history of salmon lice from four different groups, infecting salmon hosts maintained in individual tanks. Two groups (hereafter referred to as “wild lice”) were sampled from areas where there had never been any salmon farming at the time of sampling. These areas are relatively isolated from farmed areas, both geographically (by a radius of at least 200 km) and due to the outwards direction of marine currents (Oslofjord in Norway and Angus in Scotland, Heuch et al., 2000). Hence, these two groups may be assumed to be the closest possible representatives of salmon lice as they were before salmon farming started. The two other groups of lice (“farmed lice”) were sampled from salmon farms located approximately 450 km apart on the western coast of Norway (Austevoll and Frøya) where salmon farming has been taking place for about four decades, *that is* approximately 280 lice generations (Figure 1). Egg strings were collected from 38 to 50 female lice from at least 15 hosts per group, hatched in the laboratory (see e.g., Hamre, Glover, & Nilsen, 2009), and pooled together. Before the experiments all four groups of lice were bred for at least three generations in 500‐L tanks containing 15–20 naive fish in each (Industrilaboratoriet, Bergen, Norway), to reduce differences between groups of lice due to different environmental conditions at their site of origin.

### 2.2 Infection procedure

We used Atlantic salmon smolts (80–120 g) originating from the same cohort (Industrilaboratoriet, Bergen, Norway) and kept in single aquaria supplied with UV‐treated seawater with a flow rate at about 2–6 L/min, and 12 hr daylight. Fifteen fish were infected with lice from Austevoll, Frøya, and Scotland. Due to the accidental death of one fish that could not be replaced (it died the day before infection), only 14 fish were infected with lice from Oslo. Because of space limitations (laboratory rooms could not contain more than 30 individual fish tanks), we carried out this study in two different rooms, each containing one farmed and one wild group of lice (Room 1: Austevoll & Oslofjord; Room 2: Frøya & Angus). Prior to infection the fish were anesthetized with MS‐222 (75 mg/L), measured (initial length and weight), and taken back to their respective tanks for recovery. Later the same day, they were exposed to *L. salmonis* copepodites (i.e.*,* infective stages) for 1 hr, during which the water flow was stopped, the water level lowered and air was supplied directly into the tanks (as described e.g., in Mennerat et al., 2012). Due to differences in the dimensions of the tanks, water volume during infection differed (either 10 or 20 L) between the two experimental rooms. We adjusted the number of copepodites accordingly (i.e.*,* added 40 copepodites in the smaller and 80 in the bigger tanks) so that all fish were exposed to a similar density of copepodites (four copepodites per liter). Copepodites were counted using a broad‐end sterile pipette.

### 2.3 Handling of fish and lice

All fish were hand‐fed twice a day with 0.5 g of 3 mm standard industrial food pellets, following the manufacturer's recommendation. From day 40 postinfection, they were inspected daily; the number of adult females on each fish was recorded, as well as the date at which individual female lice extruded their egg strings. When all female lice on a fish had egg strings, the fish were anesthetized with 1.5 g metacaine (MS‐222) per 20 L of seawater, and all adult lice were carefully removed from the fish and placed in a cool box with seawater. Egg strings were detached from gravid females by gently pulling them with a curved forceps, after which the lice were returned to their original salmon host until the next reproductive event. The fish were gently lifted by hand so that the top of their back emerged above the surface. After placing the lice back onto the host skin, the fish were observed for a few minutes to make sure re‐attachment was successful. For each pair of egg strings, a picture of whole egg strings was taken with low magnification (3.5×) to measure total egg string length. In addition, pictures were taken with higher magnification (20×) at five distinct places along the egg string to estimate average egg length. All pictures were taken using Leica Application Suite connected to a Leica Z16APOA microscope (Leica Microsystem). This procedure was followed until day 130 postinfection, when all lice had completed their fifth reproductive event, after which the fish were euthanized with an overdose of 3.0 g of metacaine per 15 L of seawater.

### 2.4 Statistical analysis

All analyzes were performed using the *lme4* package in the statistical programming environment R 3.2.2 (http://r-project.org). All models presented here were validated by visual inspection of the normality and heteroscedasticity of residuals, and for all four of them including random effects resulted in lower AIC values than models with only fixed effects, indicating a better fit.

#### 2.4.1 Timing of reproduction

After producing their first pair of egg strings, adult female lice kept producing new pairs of egg strings at an interval of approximately 12 days (see Table 1). To test whether the timing of reproduction (i.e.*,* the dates at which female lice produce eggs, measured in days postinfection) differed between farmed and wild lice, we used a generalized linear mixed‐effects model fitted with a Poisson distribution (g*lmer*) including status (farmed vs. wild) and reproductive event (from 1 to 5) as factors. Room (1 vs. 2) and Tank (nested within Room) were used as random effect factors.

**Table 1:** Timing and fecundity of the first five reproductive events, for farmed and wild salmon lice

Reproductive event	*N*		Mean date ± *SE* (days P.I.)		Mean residual fecundity ± *SE* (corrected for parasite load)	
	Wild	Farmed	Wild	Farmed	Wild	Farmed
First	83	121	63.1 ± 0.52	65.7 ± 0.63	−147.7 ± 6.1	−118.2 ± 8.1
Second	74	112	76.4 ± 0.98	77.3 ± 0.76	58.4 ± 10.6	30.9 ± 11.0
Third	66	88	87.8 ± 0.83	89.8 ± 0.89	67.5 ± 14.4	30.6 ± 12.9
Fourth	59	73	99.1 ± 1.17	103.1 ± 0.93	58.3 ± 15.4	57.1 ± 12.5
Fifth	53	65	111.7 ± 1.20	114.6 ± 1.10	24.7 ± 18.7	41.8 ± 15.8

*N*, number of individual females; *SE*, standard error.

#### 2.4.2 Fecundity

The total number of eggs contained in each egg string was estimated by dividing total egg string length by average egg length, and fecundity was calculated as the sum for each pair of egg strings (Mennerat et al., 2012; Ugelvik, Skorping, & Mennerat, 2016). Fecundity of wild and farmed salmon lice was compared using a linear mixed‐effect model (*lmer*) with status (farmed vs. wild) and reproductive event (from 1 to 5) as factors. Because parasite load varied across fish hosts and is known to negatively affect salmon lice fecundity (Ugelvik, Mo, Mennerat, & Skorping, 2016; Ugelvik, Skorping, et al., 2016), we also included the number of female lice on each fish as a covariate. Room (1 vs. 2) and Tank (nested within Room) were used as random effect factors.

#### 2.4.3 Infection success

As a measure of infection success, we used the number of lice that reached maturity on each fish, as this variable summarizes both successful attachment and development on the host. Both male and female lice were recorded; however, male salmon lice displayed a clear tendency to jump off the fish (A. Mennerat, personal observation; Hamre & Nilsen, 2011). This resulted in male lice being washed out of the tanks at variable rates on different hosts, and we could not always ascertain whether those males actively jumped off or simply got detached as a result of death. Consequently, we only compared the infection success of females between farmed and wild lice. We used a generalized linear mixed‐effects model fitted with a binomial distribution (g*lmer*) including status (farmed vs. wild) as a factor, Room (1 vs. 2) as a random effect factor. The number of trials was defined as the number of copepodites used at infection.

#### 2.4.4 Adult mortality

To test whether adult mortality differed between farmed and wild salmon lice, we compared the number of female lice remaining on the fish after the fifth reproductive event relative to the initial number (i.e.*,* at the first reproductive event). We used a generalized linear mixed‐effects model fitted with a binomial distribution (g*lmer*) including status (farmed vs. wild) as a factor, and Room (1 vs. 2) as a random effect factor. The number of trials was defined as the initial number of adult females on the fish.

## 3 RESULTS

**Figure 2:**
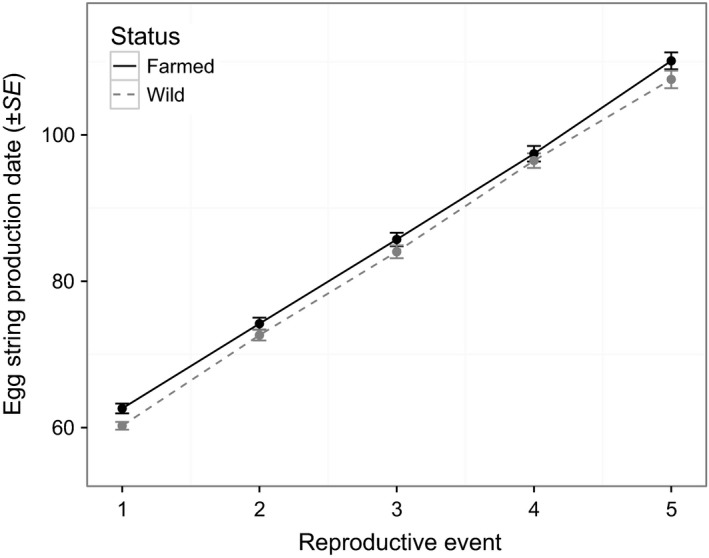
Timing of egg string production (in days postinfection) of female lice originating from either Atlantic salmon farms (“farmed”) or from unfarmed areas (“wild”), for the first five reproductive events

**Figure 3:**
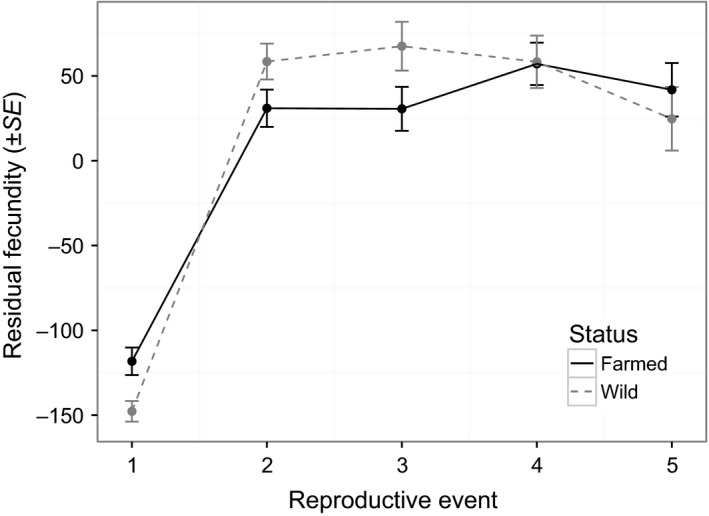
Fecundity (number of eggs produced) of female lice originating from either Atlantic salmon farms (“farmed”) or from unfarmed areas (“wild”), for the first five reproductive events. This figure represents residual fecundity after controlling for the effect of parasite load on lice fecundity (see Section 2)

**Figure 4:**
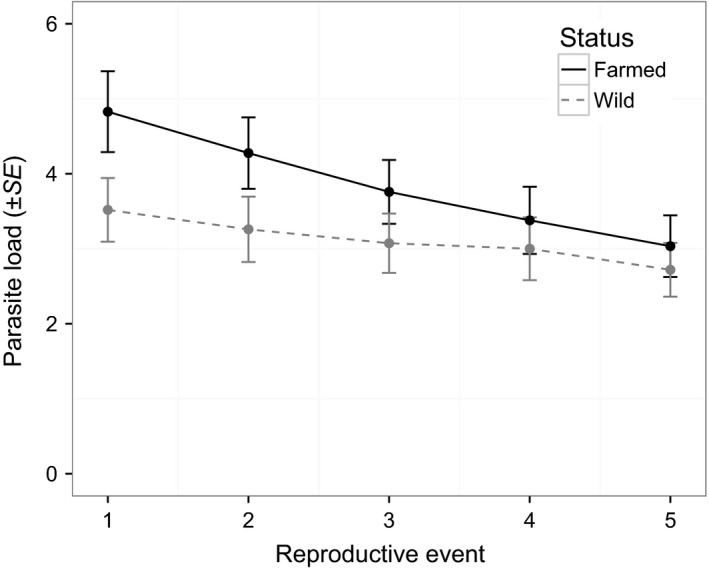
Decrease in parasite load (due to adult mortality) of female salmon lice originating from either Atlantic salmon farms (“farmed”) or from unfarmed areas (“wild”)

Farmed lice reproduced slightly (2.6/63.1 days, i.e.*,* 4%) but significantly later than wild lice (*p *= .04), and this difference did not vary over time (status * reproductive event, *p *= .22, Table 2, Figure 2). Overall fecundity was lower in farmed than in wild lice (*p *= .03), differed among reproductive events (*p *< 10^−4^), and was negatively affected by parasite load (*p *= .03, Table 3). However, fecundity was higher for farmed than for wild lice at the first reproductive event (*p *= .004), and lower at the second (*p *< 10^−4^) and third (*p *< 10^−3^) reproductive events (Table 4, Figure 3; see also Fig. S1). Farmed lice also had a higher infection success than wild lice (farmed: 0.16 ± 0.01, *N *= 27 hosts; wild: 0.12 ± 0.01, *N *= 29 hosts; *df *= 1, χ^2^ **= 5.28, *p *= .02). Finally, adult mortality was higher in farmed than in wild lice (farmed: 0.38 ± 0.06, *N *= 29; wild: 0.23 ± 0.05, *N *= 27; *df *= 1, χ^2^ **= 3.67, *p *= .055, Figure 4; see also Fig. S2).

**Table 2:** Effects of status (wild vs. farmed), reproductive event (from 1 to 5), and their interaction on the timing of reproduction of salmon lice

	Estimate	*SE*	*z*	*p*
Intercept	3.98	0.07	54.58	–
Status	−0.04	0.02	−2.06	.04
Rep. event	0.14	0.003	39.87	<10^−4^
Status x Rep. event	0.006	0.005	1.22	.22

**Table 3:** Effects of status (wild vs. farmed), reproductive event (from 1 to 5), and parasite load (number of female lice per host) on salmon lice fecundity

	Estimate	*SE*	*t*	*p*
Intercept	383.34	37.30	10.28	–
Status	33.01	14.08	2.35	.03
Rep. event	48.91	3.40	14.39	<10^−4^
Parasite load	−6.39	3.13	−2.04	.03

**Table 4:** Effect of status (wild vs. farmed) on salmon lice fecundity, for separate reproductive events. A mixed‐effect model was used, including the number of female lice on each fish as a covariate, and Room (1 vs. 2) and Tank (nested within Room) as random effect factors. Only the effects of status are reported here

Reproductive event	Estimate	*SE*	*t*	*p*
First	−32.85	10.74	8.32	.004
Second	83.34	16.50	17.56	<10^−4^
Third	83.30	20.57	12.64	<10^−3^
Fourth	22.52	25.90	0.68	.41
Fifth	25.93	29.38	0.57	.45

## 4 DISCUSSION

Compared to salmon lice sampled from unfarmed areas, those from farmed areas did not develop faster, but produced more eggs in their first clutch, and fewer eggs afterward. They achieved higher infestation intensities at maturity, but displayed higher adult mortality and hence their numbers declined more rapidly afterward. All in all, our results indicate that lice sampled from farmed areas invested more in early reproduction than lice from unfarmed areas, at the expense of later fecundity and survival.

A study by Todd, Walker, Ritchie, Graves, and Walker (2004) found no genetic differentiation between Scottish, East‐Canadian,and North‐Norwegian lice based on neutral genetic markers, and concluded that the salmon lice in the North Atlantic consists of one single panmictic population. Noticeably although, the study sample consisted almost exclusively of lice coming from areas that were either farmed or located downstream from farmed areas. The Scottish sample consisted of 11 locations on the Western coast, which has been farmed for decades, pooled together with only one location on the Eastern, nonfarmed coast. A later, more extensive survey including locations from Canada, Ireland, Shetland, Faroe Islands, and Norway found a weak, but significant genetic differentiation as well as some degree of isolation by distance (Glover et al., 2011). Neutral genetic differentiation throughout the North Atlantic is nevertheless likely weak at most, due to larval interchange between farmed and wild stocks combined with oceanic migration of wild hosts, which is assumed sufficient to prevent large, neutral genetic divergence in salmon lice in the North Atlantic. However, the story may well differ for those loci that are under selection, as even weak selection may lead to local increases in the selected alleles, as long as the populations are large enough to be free from genetic drift. Recombination would prevent such selection‐driven differentiation from being detected by microsatellite studies, unless the microsatellites used are closely linked to the genes under selection (Todd et al., 2004). Between‐population differences in characters under selection in salmon lice are largely understudied, but one recent study showed that positively selected traits such as drug resistance can rapidly spread across farms of the North Atlantic (Besnier et al., 2014). In our study, we found significant differences in life history traits (i.e.*,* traits tightly linked to fitness) between farmed and wild groups of lice that had previously been raised in the laboratory for at least three generations, indicating that those differences likely have a genetic basis. Hence, our current interpretation is that these traits may have started to differentiate due to selective changes caused by intensive salmon farming. The correlative nature of this study does, however, not allow us to determine the causes of such differentiation. Given that farmed salmon vastly outnumber wild salmon and represent a distinct genetic pool, ongoing local host adaptation might also partially explain our observations.

It is somehow unclear at this stage what consequences such adaptive changes may have in epidemiological terms, and knowledge about the life history of salmon lice is still expanding (e.g., (Ugelvik, Mo, et al., 2016; Ugelvik, Skorping, et al., 2016). From earlier studies, it seems that faster life histories correlate with higher levels of virulence (Mennerat et al., 2012), which is consistent with virulence evolution theory and more specifically the existence of a virulence—transmission tradeoff (Alizon, Hurford, Mideo, & Van Baalen, 2009; Cressler, Mc, Rozins, Van den Hoogen, & Day, 2015). One may therefore expect lice from farmed areas to display higher levels of virulence than those from unfarmed areas, and this seems to be the case (Ugelvik, Skorping, Moberg, & Mennerat, in press). However, these results remain correlative, and experimental approaches (e.g., artificial selection and/or experimental evolution) would be very useful in determining whether such apparently adaptive changes in the life history and virulence of salmon lice as well as other agricultural parasites are being caused by intensive farming.

Seen from the parasite's point of view, many of the ecological conditions that we see in farmed versus wild salmon, are also recognized in other intensive farming systems. For example, both poultry and pig farms are characterized by rapid host turnover rates, low genetic variability of hosts, and frequent use of antiparasitic drugs. While low host genetic variability may increase the speed of parasite adaptation (e.g., Altermatt & Ebert, 2008), shorter host lifespan, as well as frequent medication, reduce parasite life expectancy and thereby the prospects of future reproduction. Our main finding, of a shift to a higher investment in current reproduction, might therefore be relevant to intensive farming in general, and not just salmon farms.

## AUTHORS’ CONTRIBUTIONS

AS provided funding; AM and AS designed the study; AM, MSU, and CHJ carried out the study and collected data; AM did the statistical analysis and wrote the draft, partly based on an earlier draft written by CHJ; AS and MSU provided comments on the manuscript.

## DATA ARCHIVING STATEMENT

Data available from the Dryad Digital Repository: https://doi.org/10.5061/dryad.4db01.
