# Supplementary material for: Invest more and die faster: The life history of a parasite on intensive farms
Source: Evol Appl. 2017 Jun 21;10(9):890–6. doi: 10.1111/eva.12488 (PMC5680424; doi:10.1111/eva.12488)
Supplement: Supplementary file 1 [file EVA-10-890-s001.docx]

Figure S1. Fecundity (number of eggs produced) of female lice from either Atlantic salmon farms (Bergen and Frøya, Norway) or from unfarmed areas (Oslofjord, Norway and Angus, Scotland), for the first five reproductive events. This figure represents residual fecundity after controlling for the effect of parasite load on lice fecundity (see Methods). Fish infected with “Bergen” and “Oslo” lice were maintained in Room 1, while fish infected with “Frøya” and “Scotland” lice were maintained in Room 2.

Figure S2. Decrease in parasite load over the first five reproductive events (adult mortality) of female salmon lice originating from either Atlantic salmon farms (Bergen and Frøya, Norway) or from unfarmed areas (Oslofjord, Norway and Angus, Scotland). Fish infected with “Bergen” and “Oslo” lice were maintained in Room 1, while fish infected with “Frøya” and “Scotland” lice were maintained in Room 2.
